# Mechanical Circulatory Support as a Bridge to Transcatheter Aortic Valve Replacement in Cardiogenic Shock

**DOI:** 10.1016/j.jscai.2025.103925

**Published:** 2025-10-07

**Authors:** Ali A. Khan, Mohamed Barghout, Ahmed Elkaryoni, Hafiz M. Imran, Paul C. Gordon, Barry L. Sharaf, J. Dawn Abbott, Saraschandra Vallabhajosyula, Mohamad Alkhouli, Marwan Saad

**Affiliations:** a Warren Alpert Medical School of Brown University, Providence, Rhode Island; b Brown University Health Cardiovascular Institute, Providence, Rhode Island; c Division of Cardiology, Department of Medicine, Warren Alpert Medical School of Brown University, Providence, Rhode Island; d Department of Cardiology, Mayo Clinic College of Medicine and Science, Rochester, Minnesota

**Keywords:** cardiogenic shock, mechanical circulatory support, transcatheter aortic valve replacement, aortic stenosis

## Introduction

Transcatheter aortic valve replacement (TAVR) is an established treatment in patients with aortic stenosis (AS) and cardiogenic shock (CS), although in-hospital mortality remains high (∼10%) in this high-risk population.^1^ Severe AS with concomitant CS presents unique hemodynamic challenges—including fixed outflow obstruction and impaired left ventricular unloading—which often limit the effectiveness pharmacologic therapy, and the need for mechanical circulatory support (MCS) indicates a sicker population. Recent expert consensus from the American College of Cardiology supports the use of temporary MCS to stabilize patients with fixed cardiac output lesions (eg, AS) before definitive interventions (eg, TAVR).^2^ While temporary MCS may help improve end-organ perfusion and provide a critical bridge to definitive valve intervention, the role of MCS as a bridge to TAVR (MCS-TAVR) remains poorly examined.^3^ This study aimed to describe the national trends, utilization patterns, and in-hospital outcomes associated with MCS-TAVR in patients with AS and CS in the United States.

## Methods

The Vizient Clinical Database was used to include all patients who underwent percutaneous TAVR between January 1, 2016, and November 31, 2023, and had a concomitant diagnosis of CS during the same admission.^4^ To ensure our cohort is limited to MCS as a bridge to TAVR, patients who received MCS within 24 hours of TAVR or after TAVR were excluded. Device-specific categorization of MCS was performed, and outcomes were reported for each group. The primary end point was in-hospital all-cause mortality. All clinical comorbidities, outcomes, and interventions performed were determined by the International Classification of Diseases, Tenth Revision (ICD-10) diagnosis and procedure codes. Descriptive statistics were used to summarize baseline characteristics and outcomes, using the Wilcoxon rank-sum test and Fisher exact test where appropriate, with Stata 17.0 (StataCorp LLC) used for statistical analysis. A 2-sided *P* value of <.05 indicated statistical significance. The study was approved by the Brown University Health Institutional Review Board at the Miriam Hospital.

## Results

During the study period, 250,152 patients underwent percutaneous TAVR, of whom 6284 (2.5%) had a concomitant diagnosis of CS. Among those, 575 patients (9.2%, mean age, 70.2 ± 13.2 years; 31.3% women; 77.0% White; 6.8% Hispanic; mean Elixhauser comorbidity score, 6.5 ± 2.3) received MCS as a bridge to TAVR, that is, MCS-TAVR. Among this group, 99 patients (17.2%) underwent balloon aortic valvuloplasty in addition to MCS. A history of heart failure and coronary artery disease was present in 95.0% and 84.0% of patients, respectively. Patients were predominantly treated at large (>250 beds) teaching hospitals with high annual TAVR volume (>85/y).

The utilization of MCS-TAVR increased significantly over the study period (3.7% in 2016 vs 14.8% in 2023; *P* = .001). Intra-aortic balloon pump (IABP) was mostly used (55.3%), followed by Impella (17.9%) and extracorporeal membrane oxygenation (ECMO) (15.5%), with combined MCS device use in 11.3% of patients (IABP + ECMO, 4.4%; Impella + ECMO, 4.4%). MCS implantation was on median day 2 (IQR, 0-6 days), and TAVR was performed on median day 8 (IQR, 4-13 days) of hospitalization. The median duration between implementation of MCS and TAVR was 5 days. Among patients who underwent MCS-TAVR, the rate of in-hospital mortality was 21%, with a nonstatistically significant increase over the study period (19.1% in 2016 vs 24.7% in 2023; *P* = .56). The most common complications observed were acute kidney injury (AKI; 78.4%) and major bleeding (42.0%). The median length of stay was 20 days (IQR, 13-31 days), with a 30-day readmission rate of 13%. Table 1 summarizes in-hospital outcomes with MCS-TAVR.

**Table 1:** In-hospital outcomes of patients who underwent MCS as bridge to TAVR.

Outcomes	N = 575
Primary outcome^a^	
Death	121 (21.0)
Secondary outcomes^a^	
Conversion to SAVR	113 (19.7)
Vascular complications	63 (11.0)
Surgical vascular intervention	40 (7.0)
Aortic dissection	15 (2.6)
Aortic rupture	1 (0.2)
Pericardiac effusion/tamponade	54 (9.4)
Major amputation	3 (0.5)
Myocardial infarction	263 (45.7)
Ischemic stroke	6 (1.0)
Transient ischemic attack	6 (1.0)
Major bleeding	242 (42.0)
Acute kidney injury	451 (78.4)
Pacemaker implantation	96 (16.7)
Length of stay, d^b^	20 (13-31)
Total direct cost, $^b^	90,746 (63,945-139,216)
Disposition^c^	
Routine/home	98 (17.0)
Home health care	109 (19.0)
Skilled nursing facility	213 (37.0)
Short-term hospital	13 (2.3)
Hospice	13 (2.3)
Other/unknown	7 (1.22)
30-Day readmission	73 (12.7)
Readmission duration, d	9 (3-16)

Categorical data are N (%). Continuous variables are median (IQR).

MCS, mechanical circulatory support; SAVR, surgical aortic valve replacement; TAVR, transcatheter aortic valve replacement.

a The primary outcome of in-hospital mortality and secondary outcomes were analyzed to evaluate procedural and postprocedural complications.

b Total direct cost and length of stay are reported as median with IQR to reflect variability in health care resource utilization.

c Disposition categories were classified based on discharge location, including routine/home, home health care, skilled nursing facility, and hospice.

## Discussion

This study demonstrates the growing nationwide utilization of MCS as a bridge to TAVR in patients with AS and CS, highlighting its potential role in stabilizing the hemodynamic compromise in such sick population before definitive therapy. While multiple studies have examined the role of MCS in patients with acute myocardial infarction and CS,^5^^,^^6^ studies that evaluated MCS with TAVR were limited by the inclusion of all periprocedural MCS regardless of timing or indication, being single-center studies, or including dated cohorts from early-generation MCS and TAVR devices.^7^, ^8^, ^9^ To our knowledge, this study is the first that focused on the national utilization of MCS as a bridge to TAVR in patients with AS and CS.

The observed increase in MCS-TAVR utilization from 3.7% in 2016 to 14.8% in 2023 is notable and reflects enhanced recognition and staging of CS, growing evidence on early implementation of MCS, a greater familiarity with available devices, as well as increased physician’s experience in management of patients at high-risk for TAVR.

The in-hospital mortality in our cohort was 21%, significantly higher than that reported by Goel et al (9.9%),^1^ and is likely related to the inclusion of sicker patients requiring MCS, as opposed to all-comers with CS in the previous study. Despite the high rate of in-hospital mortality, the fact that nearly 80% of patients survived the hospitalization highlights the potential role of MCS in stabilizing those critically ill patients before TAVR. Given this is a descriptive analysis, we did not conduct propensity score matching or multivariate analysis to compare independent cohorts. Future studies with physiological data and risk adjustment are needed to assess the independent effect of MCS.

Many findings in this current study are consistent with a sick population that is liable for complications. While major bleeding typically ranges between 3% and 5%, and AKI between 4% and 7% in the general TAVR populations,^10^ we observed a significantly higher rates of major bleeding (42%), likely related to large bore access and prolonged use of anticoagulation associated with MCS, and AKI (78%) secondary to end-organ damage. In addition, only 17% of patients were discharged home, with the majority requiring skilled nursing or home health care, highlighting the substantial postdischarge morbidity, prolonged recovery, and long-term care needs among this high-risk cohort.^11^

This study has several limitations. First, given its retrospective and observational design, the potential for miscoding or undercoding of CS and MCS could not be entirely excluded. Second, the lack of granular data regarding CS SCAI staging and patient hemodynamics could have impacted our analyses. Finally, although we report device-specific utilization patterns, the lack of standardized clinical variables limits adjusted comparisons across support strategies.

## Conclusion

Mechanical circulatory support as a bridge for TAVR in patients with AS and CS is increasingly used and could provide an opportunity to improve outcomes in those high-risk population. The observed high in-hospital mortality underscores the need for careful patient selection and multidisciplinary heart team approach to guide the judicious use of MCS.
